# Early immune system alterations in patients with septic shock

**DOI:** 10.3389/fimmu.2023.1126874

**Published:** 2023-02-09

**Authors:** Huiming Tang, Shuang Qin, Zhanfei Li, Wei Gao, Manli Tang, Xijie Dong

**Affiliations:** ^1^ Trauma Center/Department of Emergency and Traumatic Surgery, Tongji Hospital of Tongji Medical College, Huazhong University of Science and Technology, Wuhan, China; ^2^ Department of Radiation Oncology, Hubei Cancer Hospital, Tongji Medical College, Huazhong University of Science and Technology, Wuhan, China

**Keywords:** sepsis, septic shock, immune system, cytokine, lymphocyte, immunoglobulin

## Abstract

This study aims to investigate the early changes in the immune systems of patients with septic shock. A total of 243 patients with septic shock were included in this study. The patients were classified as survivors (n = 101) or nonsurvivors (n = 142). Clinical laboratories perform tests of the immune system’s function. Each indicator was studied alongside healthy controls (n = 20) of the same age and gender as the patients. A comparative analysis of every two groups was conducted. Univariate and multivariate logistic regression analyses were performed to identify mortality risk factors that are independent of one another. In septic shock patients, neutrophil counts, infection biomarkers (C-reactive protein, ferritin, and procalcitonin levels), and cytokines (IL-1β, IL-2R, IL-6, IL-8, IL-10, and TNF-α) increased significantly. Lymphocyte and their subset counts (T, CD4+ T, CD8+ T, B, and natural killer cell counts), lymphocyte subset functions (the proportions of PMA/ionomycin-stimulated IFN-γ positive cells in CD4+ T cells), immunoglobulin levels (IgA, IgG, and IgM), and complement protein levels (C3 and C4) decreased significantly. Compared to survivors, nonsurvivors had higher levels of cytokines (IL-6, IL-8, and IL-10) but lower levels of IgM, complement C3 and C4, and lymphocyte, CD4+, and CD8+ T cell counts. Low IgM or C3 concentrations and low lymphocyte or CD4+ T cell counts were independent risk factors for mortality. These alterations should be considered in the future development of immunotherapies aimed at treating septic shock.

## 1 Introduction

Sepsis remains an underrecognized global health issue that causes millions of premature deaths annually and has been described as “the quintessential medical disorder of the 21st century” (1, 2). Although hundreds of clinical trials involving tens of thousands of patients and substantial funding have been conducted over the past several decades, a novel drug with a highly effective target against sepsis has yet to be developed (3). According to recent epidemiological studies, the mortality rate in sepsis remains between 25 and 30% and can reach as high as 50% in septic shock. Furthermore, most statistical data came from high-income nations. Indeed, the mortality rate is probably understated (4, 5).

Infection, the etiology of sepsis, is the root cause of a subsequent series of complex complications. Severe infection in sepsis causes immune dysfunction, which renders the body more susceptible to infection: a vicious circle (6). Immune disorders are significant contributors to sepsis’s poor prognosis. If treatment fails to restore immune function, it may have been in vain. Although organ dysfunction is temporarily alleviated, secondary bacterial infection, a vivo-derived opportunistic pathogen, and viral reactivation will attack the body and impair immune function: a new vicious cycle (3, 6). Thus, restoring immune system function is essential for the management of sepsis. Notably, septic shock is a more severe form of sepsis in which immune dysfunction is more pronounced.

The development of novel immunotherapy approaches for patients with septic shock requires a comprehensive understanding of the immune system’s extensive changes. In this study, we examined the fluctuations of immune-related markers in patients within 48 h of the onset of septic shock. On this basis, we have proposed some immunotherapy recommendations for septic shock.

## 2 Materials and methods

### 2.1 Ethical approval of the study protocol

This prospective observational cohort study was performed at Tongji Hospital (Wuhan, China). Approval was obtained from the Medical Ethics Committee of the hospital. The study was conducted in accordance with the responsible committee’s ethical standards and the Helsinki Declaration of 1975. All subjects or their legal guardians gave their written consent after being fully informed.

### 2.2 Study cohort

Patients diagnosed with septic shock within the first 48 h were recruited according to the Sepsis 3.0 criteria (7). All patients received standardized medical care following the Surviving Sepsis Campaign Guidelines (8). The exclusion criteria were as follows: (i) patients aged <18 years; (ii) patients with autoimmune diseases; (iii) patients with neoplastic or hematological diseases; (iv) patients with severe systemic inflammatory response resulting from other diseases (e.g., liver cirrhosis); and (v) patients with chronic disease requiring immunomodulatory therapy. Twenty healthy volunteers were studied alongside the patients as a control group.

### 2.3 Data collection

Demographic data, medical history, Sepsis-related Organ Failure Assessment (SOFA) score, and the Acute Physiology and Chronic Health Evaluation (APACHE) II score were obtained from the patent information system of Tongji Hospital. Subsequently, the blood routine, inflammatory biomarkers, cytokines, immunoglobulins, complement proteins, and lymphocyte subset counts and functions (the proportions of PMA/ionomycin-stimulated IFN-γ positive cells in CD4^+^ T cells) were detected and reported by the clinical laboratory. Flow cytometry analysis was performed as previously described (9)

### 2.4 Statistical analyses

Continuous and categorical variables are presented as the mean (SD), median (IQR), or number (proportion). Using the Wilcoxon rank sum test (for non-normally distributed data) or the chi-square test (for unordered categorical data), two independent samples were compared. The Kruskal–Wallis test (for non-normally distributed data) was used to compare three independent samples, followed by the Wilcoxon rank sum test. Variables associated with the risk of mortality were identified using univariate and multivariate logistic regression analyses. SPSS v26.0 (IBM, Armonk, NY) and GraphPad Prism v8.3.0 (GraphPad Software, San Diego, CA) were used to generate statistical analyses and graphs. A two-sided p-value less than 0.05 was regarded as statistically significant.

## 3 Results

### 3.1 Cohort characteristics

In total, 243 patients with septic shock were included in this study. The baseline characteristics of the study groups are shown in **Table 1**. Nonsurvivors were older and had higher APACHE II and SOFA scores than survivors. The lung was the most common infection site. Gram-negative bacteria were the most prevalent infectious agent.

**Table 1:** Demographic and clinical data of patients with septic shock.

Variable	Survivors (n=101)	Nonsurvivors (n=142)	p
Age, mean (SD), years	54.7 (11.6)	63.4 (13.8)	0.026
Male, n (%)	70 (69.3)	102 (71.3)	NS
APACHE II, mean (SD)	15.4 (5.1)	21.1 (6.9)	0.008
SOFA, median (IQR)	6.8 (5.7, 9.0)	9.4 (7.1, 13.5)	0.017
Infection site, n (%)			NS
Lung	43 (42.6)	51 (35.9)	
Abdomen	20 (19.8)	33 (23.2)	
Urinary tract	14 (13.9)	17 (12.0)	
Bone or soft tissue	8 (7.9)	12 (8.5)	
Intracranial or spinal cord	8 (7.9)	21 (14.8)	
Others	8 (7.9)	8 (5.6)	
Organism, n (%)			NS
Gram-negative	45 (44.6)	63 (44.4)	
Gram-positive	23 (22.8)	31 (21.8)	
Mixed	20 (19.8)	35 (24.6)	
Unknown	13 (12.9)	13 (9.2)	

APACHE II, Acute Physiology and Chronic Health Evaluation II; SOFA, Sepsis-related Organ Failure Assessment; mean (SD), mean (standard deviation); median (IQR), median (interquartile range); NS, not significant.

### 3.2 Blood routine results and infection biomarkers of septic shock patients showed obvious abnormalities

**Figure 1:**
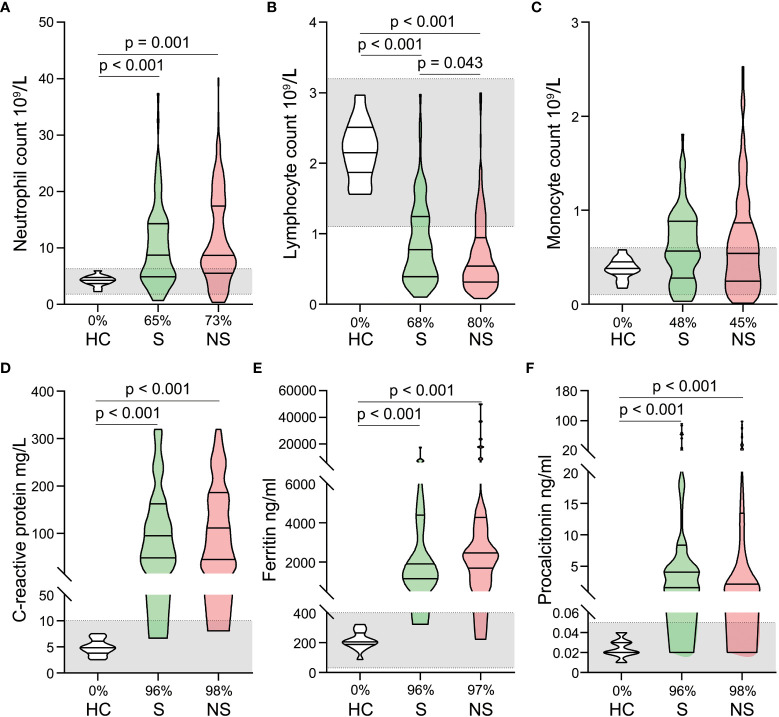
Alterations in blood routine and infection biomarkers in patients with septic shock. Neutrophil counts **(A)**, lymphocyte counts **(B)**, monocyte counts **(C)**, C-reactive protein levels **(D)**, ferritin levels **(E)**, and procalcitonin levels **(F)** were compared among healthy controls, survivors, and nonsurvivors. The shaded region represents the normal value range. The proportions of patients whose test results are above the upper limit of the normal range (**A, C–F**) or below the lower limit of the normal range **(B)** are shown on the x-axis.

Overall, blood routine (neutrophils, lymphocytes, and monocytes) and infection biomarkers (C-reactive protein, serum ferritin, and procalcitonin) were significantly different between septic shock patients and healthy controls. Neutrophil counts, C-reactive protein, serum ferritin, and procalcitonin levels were significantly elevated in patients with septic shock (**Figures 1A, D–F**), whereas lymphocyte counts were significantly reduced (**Figure 1B**). In contrast, monocyte counts did not change significantly (**Figure 1C**) and survivor and nonsurvivor analysis revealed only a difference in lymphocyte counts, which were significantly lower in nonsurvivors. (**Figure 1B**).

### 3.3 Serum cytokines levels were significantly increased in septic shock patients

**Figure 2:**
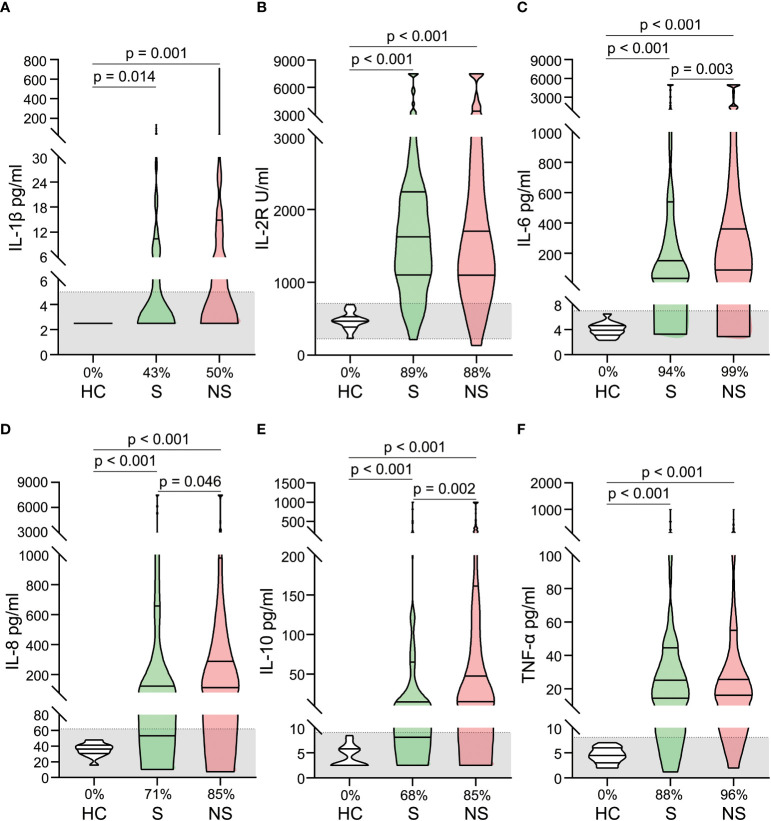
Alterations of cytokines in patients with septic shock. The levels of IL-1 β **(A)**, IL-2R **(B)**, IL-6 **(C)**, IL-8 **(D)**, IL-10 **(E)**, and TNF- α **(F)** were compared among healthy controls, survivors, and nonsurvivors. The shaded region represents the normal value range. The proportions of patients whose test results are above the upper limit of the normal range are shown on the x-axis **(A–F)**.

In patients with septic shock, pro- and anti-inflammatory cytokines increased dramatically, a phenomenon known as a cytokine storm. Significantly elevated serum levels of IL-1β, IL-2R, IL-6, IL-8, IL-10, and TNF-α were observed. Some cytokine levels were hundreds of times that of healthy controls (**Figures 2A–F**). When survivors and nonsurvivors were compared, nonsurvivors had significantly higher levels of IL-6, IL-8, and IL-10 (**Figures 2C–E**).

### 3.4 Serum immunoglobulin and complement levels significantly decreased in septic shock patients

**Figure 3:**
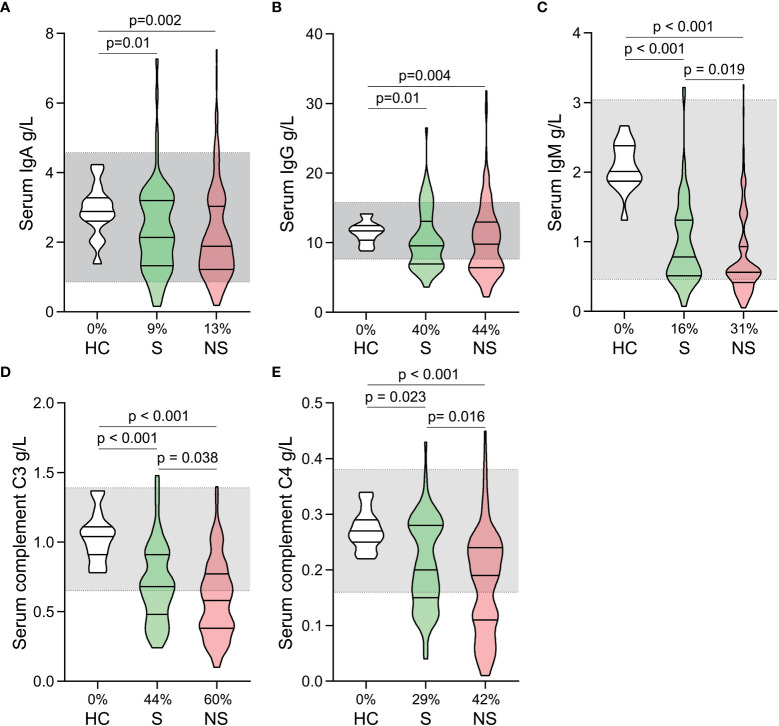
Fluctuations of serum immunoglobulin and complement levels in patients with septic shock. The levels of IgA **(A)**, IgG **(B)**, IgM **(C)**, C3 **(D)**, and C4 **(E)** were compared among healthy controls, survivors, and nonsurvivors. The shaded region represents the normal value range. The proportions of patients whose test results are below the lower limit of the normal range are shown on the x-axis **(A–E)**.

Both survivors and nonsurvivors had significantly decreased levels of serum immunoglobulin (IgA, IgG, and IgM) and complement (C3 and C4) compared to healthy controls. Although many patients’ levels of immunoglobulin and complement are still within the normal range, they are generally lower than those of healthy controls (**Figures 3A–E**). When survivors and nonsurvivors were compared, nonsurvivors had significantly lower levels of IgM, C3, and C4 (**Figures 3C–E**).

### 3.5 Lymphocyte subset counts significantly decreased in septic shock patients

**Figure 4:**
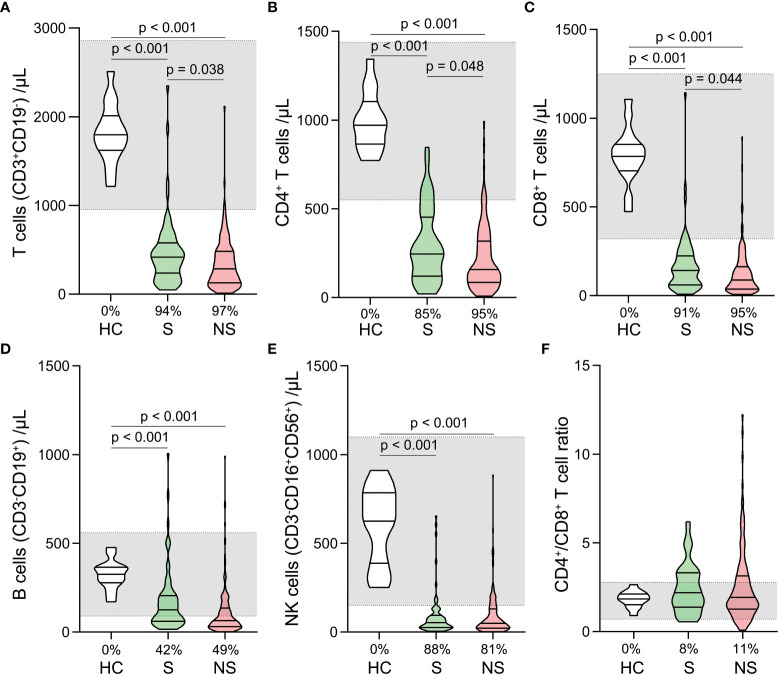
Changes of lymphocyte subset counts and CD4^+^/CD8^+^ ratios in septic shock patients. The counts of T cells **(A)**, CD4^+^ T cells **(B)**, CD8^+^ T cells **(C)**, B cells **(D)**, Natural killer (NK) cells **(E)**, and CD4^+^/CD8^+^ ratios **(F)** were compared among healthy controls, survivors, and nonsurvivors. The shaded region represents the normal value range. The proportions of patients whose test results are below the lower limit of the normal range are shown on the x-axis **(A–F)**.

Total T cell, CD4^+^ T cell, CD8^+^ T cell, B cell, and Natural killer (NK) cell counts were significantly lower in survivors and nonsurvivors than in healthy controls (**Figures 4A–E**). There was no significant difference among the three groups in CD4^+^/CD8^+^ T cell ratios (**Figure 4F**). When survivors and nonsurvivors were compared, the counts of T cells, CD4^+^ T cells, and CD8^+^ T cells were significantly lower in nonsurvivors (**Figures 4A–C**).

### 3.6 Impairment of CD4^+^ T cell function in septic shock patients

**Figure 5:**
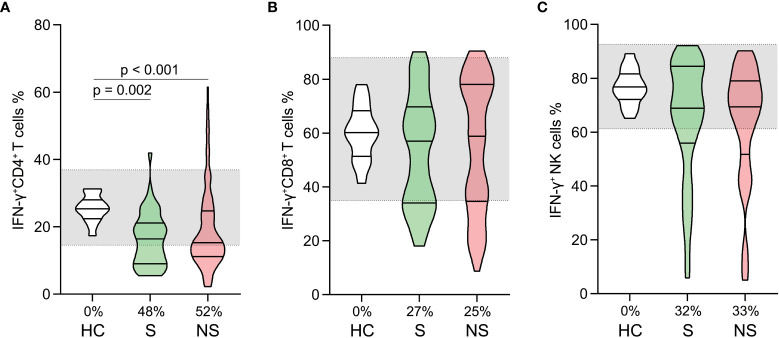
Changes in lymphocyte functions in patients with septic shock. The proportions of PMA/ionomycin-stimulated IFN-γ positive cells in CD4^+^ T cells **(A)**, CD8^+^ T cells **(B)**, and Natural killer (NK) cells **(C)** were compared among healthy controls, survivors, and nonsurvivors. The shaded region represents the normal value range. The proportions of patients whose test results are below the lower limit of the normal range are shown on the x-axis **(A–C)**.

We analyzed the proportions of PMA/ionomycin-stimulated IFN-γ positive cells within CD4^+^, CD8^+^ T cells, and NK cells. The proportions of IFN-γ positive CD4^+^ T cells were lower in survivors and nonsurvivors compared to healthy controls (**Figure 5A**). No comparable outcomes were observed for CD8^+^ T and NK cells (**Figures 5B, C**). Moreover, the proportions of IFN-γ positive CD4^+^, CD8^+,^ and NK cells did not differ significantly between survivors and nonsurvivors (**Figures 5A–C**).

### 3.7 Analysis of risk factors related to death in septic shock patients

Based on the above findings, we determined that there were significant differences between survivors and nonsurvivors in several indicators. For these indicators, a death-related risk factor analysis was conducted (**Table 2**). After adjusting for confounding variables, we found that SOFA, APACHE II scores, lymphocyte counts, IgM levels, C3 levels, and CD4^+^ T cell counts were significantly correlated with mortality.

**Table 2:** Univariate and multivariate logistic regression analysis.

Variable	Univariate analysis		Multivariate analysis	
	OR (95% CI)	*p*	OR (95% CI)	*p*
Age	1.08 (1.01, 1.17)	0.022	0.99 (0.96, 1.10)	NS
SOFA score	1.40 (1.22, 1.61)	<0.001	1.35 (1.10, 1.48)	0.017
APACHE II score	1.24 (1.17, 1.32)	<0.001	1.25 (1.15, 1.34)	<0.001
Lymphocyte counts	0.59 (0.37, 0.93)	0.013	0.67 (0.46, 0.98)	0.047
IL-6 levels	1.05 (1.01, 1.12)	0.018	1.02 (0.97, 1.07)	NS
IL-8 levels	1.00 (1.00, 1.00)	NS		
IL-10 levels	1.09(1.03, 1.23)	0.026	1.05 (0.96, 1.16)	NS
IgM levels	0.56 (0.34, 0.90)	0.017	0.62 (0.41, 0.99)	0.044
C3 levels	0.26 (0.10, 0.73)	0.010	0.31 (0.15, 0.84)	0.028
C4 levels	0.02 (0.01 0.39)	0.011	0.04 (0.02, 0.46)	NS
T cell counts	0.93 (0.90, 1.00)	0.020	0.97 (0.92, 1.08)	NS
CD4^+^ T cell counts	0.90 (0.88, 0.99)	0.009	0.93 (0.91, 1.00)	0.023
CD8^+^ T cell counts	0.98 (0.96, 1.00)	NS		

APACHE, Acute Physiology and Chronic Health Evaluation; SOFA, Sepsis-related Organ Failure Assessment; OR, odds ratio; CI, confidence interval; NS, not significant.

## 4 Discussion

In this study, we comprehensively analyzed immune system changes in patients with septic shock. We found inflammatory stimulation existed in septic shock patients including increased neutrophil counts, activation of the complement system, and cytokine storm. Immunosuppression also existed including lymphocyte depletion, decreased antibody levels secreted by B cells, and impaired CD4^+^ T cell functions. These results provide a theoretical foundation for assessing the prognosis of patients with septic shock and exploring immunotherapy.

We observed significant increases in infection biomarkers and neutrophil counts in patients with septic shock. Normally, the half-life of circulating neutrophils is brief (7–12 h *in vivo*). However, the decreased sensitivity of neutrophils to apoptosis during sepsis significantly lengthened their lifespan, which may be one of the reasons for their increase (10–12). Neutrophils: a double-edged sword in sepsis (13). They have potent phagocytic activity and serve as the “front line” of defense against pathogenic microorganisms (14). However, lysosomal enzymes released by neutrophils can damage blood vessels and surrounding tissues, thereby increasing the likelihood of organ failure (15, 16). During sepsis, neutrophils exhibited obvious abnormalities, including reduced ROS production, increased immature neutrophils, impaired migration and bacterial clearance, and cytokine secretion disorder. All of these changes were associated with an increased mortality risk in sepsis (17–19). Consequently, excessive abnormal neutrophil consumption may aid in enhancing the prognosis of septic shock. An earlier study supported the notion that neutrophil consumption ameliorated LPS-induced systemic inflammation and liver injury in a mouse model (20).

Consistent with previous studies, we found that the serum cytokine levels of septic shock patients significantly increased and were characterized by cytokine storms (21). Moreover, studies have shown that elevated levels of IL-6, IL-8, and IL-10 are strongly associated with mortality (21–23), which is consistent with our findings. For many years, an excessive systemic inflammatory response was believed to contribute to sepsis-related death. In light of the general increase in serum cytokines, it has been suggested that inhibiting the inflammatory response to prevent cytokine storm syndrome could aid in reducing mortality. However, this method failed to significantly improve the prognosis. In some instances, this method may even worsen the prognosis (21, 24, 25). In this study, we found that although the levels of multiple cytokines increased in nonsurvivors, they were not risk factors for mortality. Therefore, cytokine storms in sepsis may be merely a biological marker of disease severity and not the cause of organ damage (26). Cytokines play an important role in the maintenance of the innate immune response and efficient pathogen clearance. Moreover, during sepsis, most inflammatory mediators exhibit pleiotropic effects on the downstream pathway and interdependent biological activities (27). Inhibiting the innate immune response to reduce elevated cytokine levels should therefore be approached with caution.

We discovered that nonsurvivors had lower IgM levels than survivors. In addition, logistic regression analysis revealed that low IgM levels were a risk factor for mortality, indicating that IgM may play an important role in prognosis. Furthermore, numerous studies have shown that IgM levels are closely related to the prognosis of sepsis and have a significant protective effect in sepsis (28, 29). In clinical trials, receiving intravenous immunoglobulin containing only IgG (IVIG) did not demonstrate a clear benefit (30–32). However, the use of IgM-enriched immunoglobulin (IgGAM) appears to be an effective strategy. A meta-analysis of 19 clinical trials involving over 1,500 patients with sepsis evaluated the efficacy of IgGAM and revealed that its use could significantly reduce mortality (33). This observation was consistent with several other studies (34, 35). Therefore, we propose that IgM supplementation or the prevention of their decline merits further study. In addition, we observed a decline in serum complement C3 and C4 levels in septic shock patients, with the decrease being more pronounced in nonsurvivors. Moreover, lower C3 levels were found to be a risk factor for mortality, indicating that excessive complement system activation led to increased C3 and C4 consumption and was associated with patient outcomes. This is consistent with previous findings that sepsis-induced complement system activation causes endothelial dysfunction, coagulopathy, and cardiovascular abnormalities (36, 37). Inhibiting complement system activation was beneficial in sepsis (38–40). The complement levels (especially the C3 levels) may be a significant biomarker for prognostic stratification, as indicated by our findings, which support and expand upon previous research.

Lymphocyte depletion and dysfunction are important causes of immunosuppression (41–43). In this study, we observed a dramatic decrease in the lymphocyte, T cell, CD4^+^ T cell, CD8^+^ T cell, B cell, and NK cell counts of patients with septic shock. Similarly, the proportion of INF-γ positive CD4^+^ T cells decreased dramatically. Early immunosuppression due to lymphocyte compartment abnormalities was detected in septic shock patients, as indicated by these results. In addition, survivors and nonsurvivors had different lymphocyte, T cell, CD4^+^ T cell, and CD8^+^ T cell counts, and low CD4^+^ T cell counts were identified as a risk factor for mortality. Due to their capacity to interact with innate immune cells and adaptive immune cells, lymphocytes play a crucial role in the anti-infection immune response (44, 45). As described previously, excessive lymphocyte depletion worsens sepsis prognosis. In numerous animal models of sepsis, reducing lymphocyte depletion had positive effects (46, 47). In contrast to rapid and transient cytokine storms, immunosuppression caused by abnormal lymphocyte counts and function is typically long-lasting, progressive, and eventually fatal (48). Therefore, finding solutions for lymphocyte depletion may contribute to relieving immunosuppression and thus benefit septic shock patients.

This study has several limitations that should be noted. Firstly, although all data were collected within 48 h after the patient was diagnosed with septic shock, the results would be more convincing if the time range was reduced due to significant fluctuations in the immune system. Moreover, our study focused on a one-time point, and the inclusion of data at multiple time points would make a clearer description of immune system changes. Lastly, this is a prospective single-center study. The nature of our study determined our findings need to be verified by large, multi-center studies of rigorous design.

## 5 Conclusions

Significant immune system alterations occurred in patients with septic shock: Neutrophil counts, infection biomarkers, and cytokine levels increased significantly; Lymphocyte counts, immunoglobulin and complement protein levels, and lymphocyte subset counts declined dramatically; CD4^+^ T cell function decreased. In nonsurvivors, the levels of IL-6, IL-8, and IL-10 were greater than in survivors, whereas the levels of IgM, complement C3 and C4, and the counts of lymphocytes, T cells, CD4^+^ and CD8^+^ T cells were the opposite. Mortality risk factors include low lymphocyte counts, IgM levels, C3 levels, and CD4^+^ T cell counts. Suppressing the cytokine storm *via* the inhibition of the immune response should be approached with caution. Immunotherapy for eradicating excessively dysfunctional neutrophils, supplying IgM, inhibiting the overactivation of the complement system, and reducing lymphocyte depletion merits additional research.

## Data availability statement

The raw data supporting the conclusions of this article will be made available by the authors, without undue reservation.

## Ethics statement

The studies involving human participants were reviewed and approved by Medical Ethics Committee of Tongji Hospital of Tongji Medical College, Huazhong University of Science and Technology. The patients/participants provided their written informed consent to participate in this study.

## Author contributions

XD and MT conceptualized and designed the study. HT and SQ acquired the data. HT, SQ, ZL, and WG analyzed and interpreted the data. HT and SQ drafted the article. ZL, WG, XD, and MT critically revised the manuscript. HT and SQ contributed equally to this work. XD and MT share senior authorship. All authors contributed to the article and approved the submitted version.
